# Exposure to Zinc Sulfate Results in Differential Effects on Olfactory Sensory Neuron Subtypes in Adult Zebrafish

**DOI:** 10.3390/ijms17091445

**Published:** 2016-08-31

**Authors:** James T. Hentig, Christine A. Byrd-Jacobs

**Affiliations:** Department of Biological Sciences, Western Michigan University, 1903 W Michigan Ave, Kalamazoo, MI 49008, USA; james.t.hentig.1@nd.edu

**Keywords:** zebrafish, zinc sulfate, olfactory epithelium, scanning electron microscopy, cilia, microvilli, sensory neurons, amino acids, bile salts, behavior

## Abstract

Zinc sulfate is a known olfactory toxicant, although its specific effects on the olfactory epithelium of zebrafish are unknown. Olfactory organs of adult zebrafish were exposed to zinc sulfate and, after 2, 3, 5, 7, 10 or 14 days, fish were processed for histological, immunohistochemical, ultrastructural, and behavioral analyses. Severe morphological disruption of the olfactory organ was observed two days following zinc sulfate exposure, including fusion of lamellae, epithelial inflammation, and significant loss of anti-calretinin labeling. Scanning electron microscopy revealed the apical surface of the sensory region was absent of ciliated structures, but microvilli were still present. Behavioral analysis showed significant loss of the ability to perceive bile salts and some fish also had no response to amino acids. Over the next several days, olfactory organ morphology, epithelial structure, and anti-calretinin labeling returned to control-like conditions, although the ability to perceive bile salts remained lost until day 14. Thus, exposure to zinc sulfate results in rapid degeneration of the olfactory organ, followed by restoration of morphology and function within two weeks. Zinc sulfate appears to have a greater effect on ciliated olfactory sensory neurons than on microvillous olfactory sensory neurons, suggesting differential effects on sensory neuron subtypes.

## 1. Introduction

The peripheral olfactory organs, unlike the peripheral structures of most other sensory systems, are openly exposed to the environment, allowing exposure to a wide variety of toxicants. Perhaps for this reason, the olfactory system is renowned for its natural neuronal turnover [1] and ability to restore critical functions related to nutrition, protection, and reproduction. Fish, in particular, are adept at adult neurogenesis and neural regeneration [2,3].

Fish olfactory organs are vulnerable to pollution and contaminants in their home waters since they are directly exposed to the aquatic environment. The deleterious effects of a wide variety of toxicants on the olfactory epithelium of fish have been well studied (see [4] for a review). A series of experiments investigating the effects of zinc sulfate and a variety of other chemicals on the catfish olfactory epithelium showed that, while some degree of degradation of the olfactory epithelium was observed among all salt solutions and heavy metals examined, zinc sulfate was found to cause the most severe damage [5]. Irrigation of the olfactory organ with various concentrations of zinc sulfate produced degeneration of olfactory sensory neurons (OSNs), although the tissue regenerated within a week. More significant effects on olfactory organ structure were seen with prolonged exposure to the toxicant, and little regeneration occurred.

The zebrafish (*Danio rerio*) is a long-standing model widely used in developmental and neuroplasticity studies, in part because of its rapid rate of recovery from damage [6]. These features, as well as their low cost and small size, make the zebrafish olfactory system an excellent model in which to examine toxicology [7,8,9]. The zebrafish olfactory system consists of two olfactory rosettes, with olfactory nerves that project to olfactory bulbs in the rostral-most part of the brain. The rosettes comprise lamellae containing regions of non-sensory epithelium and olfactory epithelial tissue, a cell-dense pseudostratified columnar epithelium with a variety of cell types [10,11]. The olfactory epithelium consists of basal cells, supporting cells, and olfactory sensory neurons.

Zebrafish and other teleosts possess three types of OSNs dispersed throughout the sensory epithelium: Ciliated OSNs, microvillous OSNs, and crypt neurons [12]. These OSN subtypes display distinct physiological and morphological differences. Ciliated OSNs respond to bile salts [13] that provide social cues [14,15], and these cells have a thin dendrite possessing cilia [11,12]. Microvillous OSNs respond to amino acids and nucleotides [16] that initiate feeding behaviors [13,14,15], and the cells have a thick dendrite with microvilli emanating from the dendritic knob [12]. Crypt OSNs are oblong in shape, with both cilia and microvilli that are submerged in the cell body, and are found near the apical surface of the sensory epithelium [11,12]. While their ligands are unknown, it is believed that crypt neurons may perceive pheromones involved in reproduction [17,18].

Previous work in zebrafish has shown that intranasal irrigation with Triton X-100 causes rapid degeneration of the olfactory epithelium, with complete regeneration following a short recovery period [19]. Olfactory-mediated behavior is affected, with fish retaining the ability to sense amino acids but temporarily losing the ability to respond to bile salts [20,21]. These results appear to indicate a differential response of OSN subtypes to detergent exposure. The current study aimed to determine whether this susceptibility of ciliated OSNs and resistance of microvillous OSNs was a universal response to chemical damage by examining the effects of intranasal irrigation with zinc sulfate through analysis of histology, immunohistochemistry, scanning electron microscopy, and behavior. We hypothesized that zinc sulfate exposure would result in rapid neuronal degeneration and regeneration in the lesioned olfactory organs, with loss of ciliated OSNs and the ability to perceive bile salts and retention of microvillous OSNs and amino acid perception. We found that exposure to zinc sulfate does differentially affect olfactory sensory neuron subtypes in adult zebrafish.

## 2. Results

### 2.1. Time Course of Degeneration and Regeneration of the Zebrafish Olfactory Epithelium Following Exposure to Zinc Sulfate

**Figure 1:**
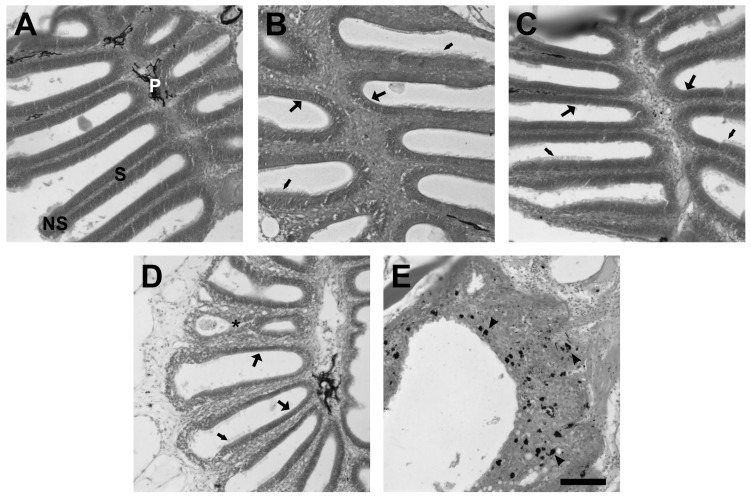
The effects of various doses of zinc sulfate applied intranasally were examined 3 days after exposure in hematoxylin and eosin-stained horizontal sections. (**A**) Unlesioned control olfactory organs had sensory (S) and non-sensory (NS) regions lining the lamellae and pigment cells (P) dispersed throughout the lamina propria. There were no obvious effects of 0.2 M (**B**) or 0.5 M (**C**) zinc sulfate. At both doses, the sensory (large arrows) and non-sensory (small arrows) epithelia had a uniform appearance. There were effects apparent when the olfactory organs were exposed to 1 M zinc sulfate (**D**). Although the non-sensory epithelium (small arrow) looked unaffected, the sensory epithelium (large arrows) appeared thin and was occasionally fused (asterisk). The 5 M zinc sulfate concentration had an even more disruptive effect on the tissue (**E**). Very little structure remained and it was not possible to distinguish sensory and non-sensory epithelia. Pigment (arrowheads) was dispersed throughout the disorganized tissue. Scale bar = 50 µm (**A**–**E**).

Morphological observations of fish olfactory organs treated with various doses of zinc sulfate allowed exploration of a dose-response relationship. Olfactory organs were exposed to 0.2 M, 0.5 M, 1 M, or and 5 M zinc sulfate through intranasal irrigation and examined after three days. Hematoxylin and eosin-stained sections were examined for general appearance and structural changes. In unlesioned control olfactory organs, lamellae are lined with sensory epithelium exhibiting typical pseudostratified columnar morphology and non-sensory epithelium that had a columnar morphology with long cilia extending from the apical surface (Figure 1A). Pigment cells were present in the lamina propria. There were no obvious deleterious effects on lamellar or epithelial morphology at 0.2 and 0.5 M (Figure 1B,C). Both sensory and non-sensory regions had a typical appearance, at least at the light-microscopic level. Exposure to 1 M zinc sulfate resulted in noticeable alterations in epithelial structure (Figure 1D). The sensory epithelium appeared thin, and some lamellae were fused. Following application of 5 M zinc sulfate, the olfactory organ was severely destroyed, with only little tissue remaining, no evidence of typical lamellar structure, numerous vacuoles present throughout the remaining tissue, and fragmentation of the pigment cells (Figure 1D). As a result of these observations, 1 M zinc sulfate was selected for this study, as it was the lowest dose that resulted in an obvious effect.

**Figure 2:**
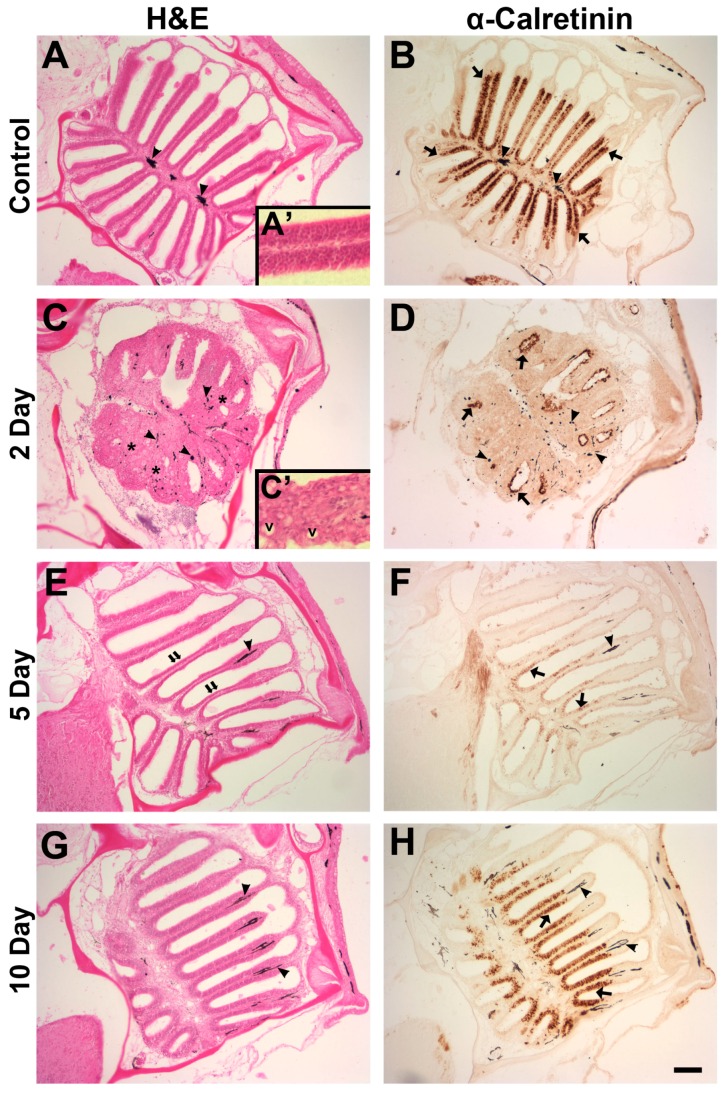
The morphology of the olfactory organ and distribution of olfactory sensory neurons were examined with histological and immunohistochemical techniques. Unlesioned control olfactory organs sectioned in the horizontal plane displayed a semi-symmetrical shape with radiating lamellae (**A**) and typical olfactory epithelium appearance at higher magnification (**A′**); there was dense anti-calretinin labeling along the sensory epithelium ((**B**), arrows). Pigment cells were apparent in the lamina propria (arrowheads); (**C**) olfactory organs 2 days after intranasal infusion with 1 M zinc sulfate exhibited inflammation and fusion of lamellae (asterisks), and pigment was more dispersed (arrowheads). (**C′**) Higher magnification revealed vacuoles (v) and a generally disorganized epithelium; (**D**) anti-calretinin labeling (arrows) was diminished and confined to the apical surface of the epithelium. By 5 days after 1 M zinc sulfate exposure, the sensory epithelium was noticeably thinner than control tissue ((**E**), double arrows), and anti-calretinin labeling showed there were numerous olfactory sensory neurons dispersed throughout the tissue ((**F**), arrows). Pigment cells were again confined to the lamina propria (arrowheads). The morphology of the olfactory organ 10 days after 1 M zinc sulfate application more closely resembled that of control (**G**), and anti-calretinin labeling appeared similar to control levels in amount and intensity ((**H**), arrows). Scale bar = 100 μm (**A**–**H**) or 25 µm (**A′**,**C′**).

A time course of effects of 1 M zinc sulfate was examined by staining adjacent sections with hematoxylin and eosin and anti-calretinin. Anti-calretinin is a marker that is used to label mature OSNs, although it is debated whether both ciliated and microvillous OSNs express the antigen [13,22,23,24,25]. For our purposes, this label was used to denote general OSN distribution. Unlesioned control organs were symmetrical with lamellae projecting out from the central raphe (Figure 2A), and the olfactory epithelium had a typical appearance (Figure 2A’). Control olfactory organs had heavy anti-calretinin labeling of densely packed OSNs throughout the sensory epithelium and no labeling in the non-sensory region, as expected (Figure 2B). Two days following exposure to zinc sulfate, there was severe morphological disruption of the olfactory organ (Figure 2C), and little anti-calretinin labeling remained (Figure 2D). The organ had some anatomical resemblance to control tissue, but there was obvious inflammation of the epithelium. Lamellae of lesioned organs appeared fused and large vacuoles and dispersed pigment could be seen within the swollen epithelium (Figure 2C’). Anti-calretinin labeling was minimal and was found only in isolated pockets, primarily at the apical portion of the epithelium. By five days after exposure, inflammation appeared to have decreased, allowing for a closer resemblance to control morphology, although the sensory epithelium appeared thin (Figure 2E). Pigment cells were again localized to the lamina propria. Anti-calretinin labeling had returned sparsely throughout the olfactory epithelium five days after exposure to the toxicant (Figure 2F). When permitted 10 days recovery following zinc sulfate administration, the anatomical structure of the rosette appeared fully intact, and the epithelium had regained a control-like appearance (Figure 2G). Anti-calretinin labeling showed densely packed OSNs throughout the sensory epithelium (Figure 2H) and resembled that of control tissue.

**Figure 3:**
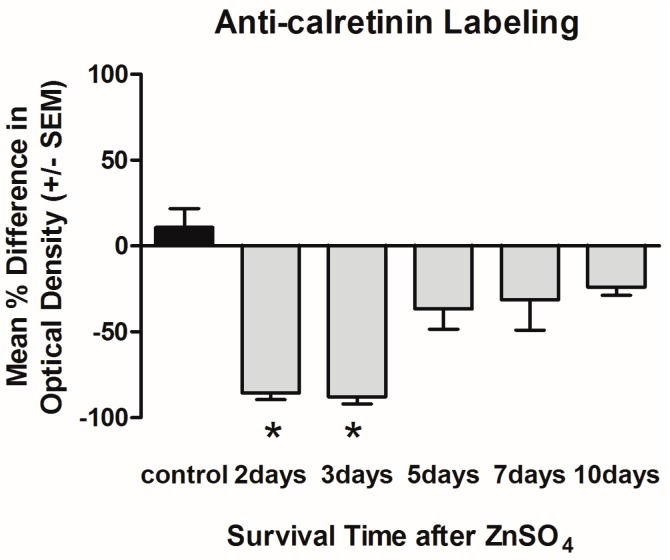
Anti-calretinin immunoreactivity was compared between treated and internal control olfactory organs using the mean percent difference in optical density. There was a significant decrease in anti-calretinin labeling at 2 and 3 days after zinc sulfate irrigation, compared to unlesioned control fish. By 5 days after chemical exposure, the amount of anti-calretinin labeling was not different from controls, and this continued at 7 and 10 days. * *p* < 0.05.

Densitometry was used to quantify the levels of anti-calretinin labeling through the degeneration and regeneration time course, as a means of estimating the quantity of OSNs in the treated olfactory organ compared to internal control sides. Unlesioned control fish had 10.8 ± 10.9 (mean ± SEM) percent difference in staining between the right and the left olfactory organs (Figure 3), showing that there was some variation in amount of label between right and left olfactory organs under control conditions, although the value was not significantly different from zero (*t*-test, *p* = 0.43). On the lesioned side at two days following intranasal irrigation with zinc sulfate, anti-calretinin labeling had significantly decreased to −85.6% ± 3.7% compared to control fish (ANOVA, *p* < 0.05; Figure 3). By three days, the mean percent difference was similar (−87.9% ± 4.1% and was also significantly different from control values (ANOVA, *p* < 0.05; Figure 3). At both two and three days, the right olfactory organs had significantly less anti-calretinin staining than the left, internal control organs (2-way ANOVA, *p* < 0.05). At five days after exposure to the toxicant, anti-calretinin labeling appeared somewhat diminished (−36.7% ± 11.8%; Figure 3), although the mean percent difference was not different from zero (*t*-test, *p* = 0.09). By seven days after zinc sulfate exposure, anti-calretinin was slightly decreased (−31.3% ± 17.7%; Figure 3) and continued to move towards control levels. The presence of anti-calretinin labeling resembled control levels more closely by 10 days after treatment (−24.26% ± 4.67%; Figure 3); the percent difference in optical density of antibody labeling had not returned completely to that of unlesioned control fish, but was not significantly different from control values (ANOVA, *p* > 0.05).

### 2.2. Ultrastructural Analysis of Epithelial Surface with Scanning Electron Microscopy

**Figure 4:**
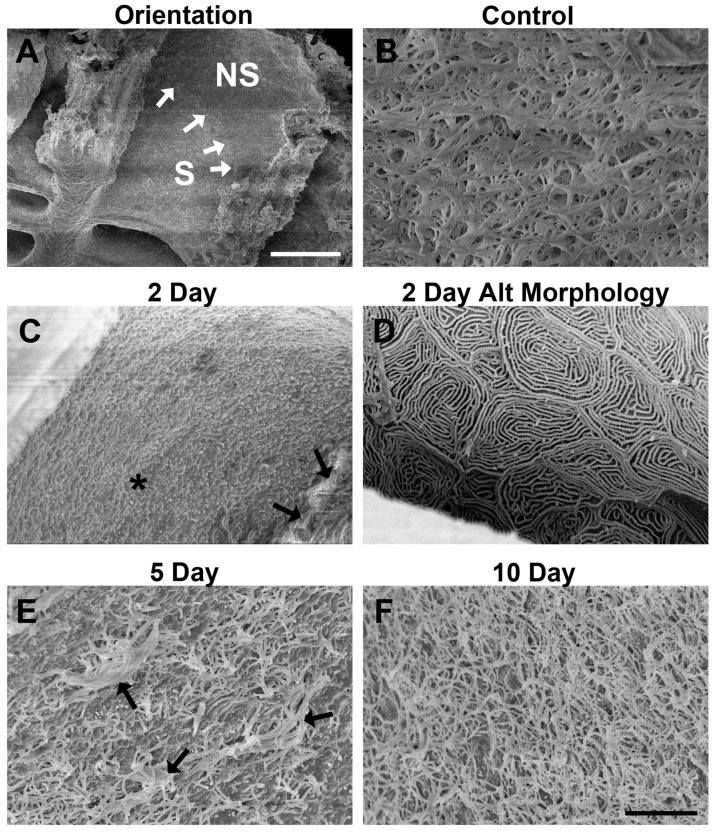
Scanning electron microscopy allowed analysis of the effects of zinc sulfate exposure on surface structures. (**A**) The sensory (S) and non-sensory (NS) regions of a lamella are distinguished with a defined separation (white arrows) in control fish; (**B**) the surface of control sensory epithelia was densely packed with cilia from OSNs, which obscured viewing of the shorter microvilli that are also present on the apical surface; (**C**) at 2 days after zinc sulfate exposure, the sensory epithelial surface appeared to contain only microvilli, with no evidence of cilia (*). Cilia in the non-sensory epithelium remained (black arrows); (**D**) an alternate morphology with no cilia or microvilli was seen at 2 days in 25% of the specimens examined. In these specimens, only microridges were apparent; (**E**) on the surface of the sensory epithelium of fish examined 5 days after infusion with the toxicant, intermittent cilia (black arrows) were present across the mat of microvilli; (**F**) by 10 days of recovery, the sensory epithelium appeared to be densely packed with cilia and microvilli, similar to control tissue. Scale bar in (**A**) = 100 μm; scale bar in (**F**) = 7 μm (**B**–**F**).

Lamellae of olfactory organs from control and lesioned fish were examined with scanning electron microscopy from an en face view. In unlesioned control olfactory organs, clear separations of sensory and non-sensory regions were observed as an evident ridge formed by distinctly different ciliated structures (Figure 4A). The non-sensory epithelium possessed cilia that were considerably longer in comparison to sensory cilia. The sensory epithelium of unlesioned organs displayed densely packed mats of ciliated OSNs covering the surface (Figure 4B). Two days following zinc sulfate exposure, ciliated structures in the sensory regions were no longer present, and microvilli were seen throughout the apical surface (Figure 4C). Based on observation of the surface structure, it appeared that the sensory epithelium contained only microvillous OSNs and there was a complete absence of sensory ciliated structures. It is relevant to note that following exposure, cilia in the non-sensory region appeared to be undamaged (Figure 4C). An alternative morphology was infrequently observed, in approximately 25% of the samples. In these specimens, the olfactory organ appeared barren with irregularly shaped concentric circles with raised ridges formed across the entire organ (Figure 4D). These appeared to resemble the microridges of the zebrafish epidermis and non-sensory epithelium described by Hansen and Zeiske [1]. Similar structures were described as microridges of supporting cells following mercuric chloride olfactory toxicity in the carp *Labeo rohita* [26]. When examined five days following exposure to zinc sulfate, cilia were now observed again in the sensory region, although they appeared thinner, shorter, and in considerably less quantity compared to control tissue (Figure 4E). When the olfactory organ was given 10 days to recover from toxicant exposure, the sensory regions were again densely covered in ciliated structures (Figure 4F), as in unlesioned control fish.

### 2.3. Effects on Olfactory-Mediated Behavior

Before testing behavioral responses to odor mixtures, fish were given time to acclimate to the testing tank, during which time all fish exhibited various typical behaviors, as described by the Zebrafish Behavior Catalog (ZBC) [27], including darting (ZBC term 1.41) and exploratory behavior (ZBC term 1.54). After the fish acclimated to the tank, they began demonstrating general swimming behavior: exhibiting steady speed, absence of rapid movements, and making occasional turns. Water was administered simultaneously through both tubes of the testing apparatus to six unlesioned control fish to test for changes in turning behavior as a result of disturbance of the tank water; this mechanical control showed there was no significant change in turning behavior in the absence of odor (data not shown, *p* = 28.56).

**Figure 5:**
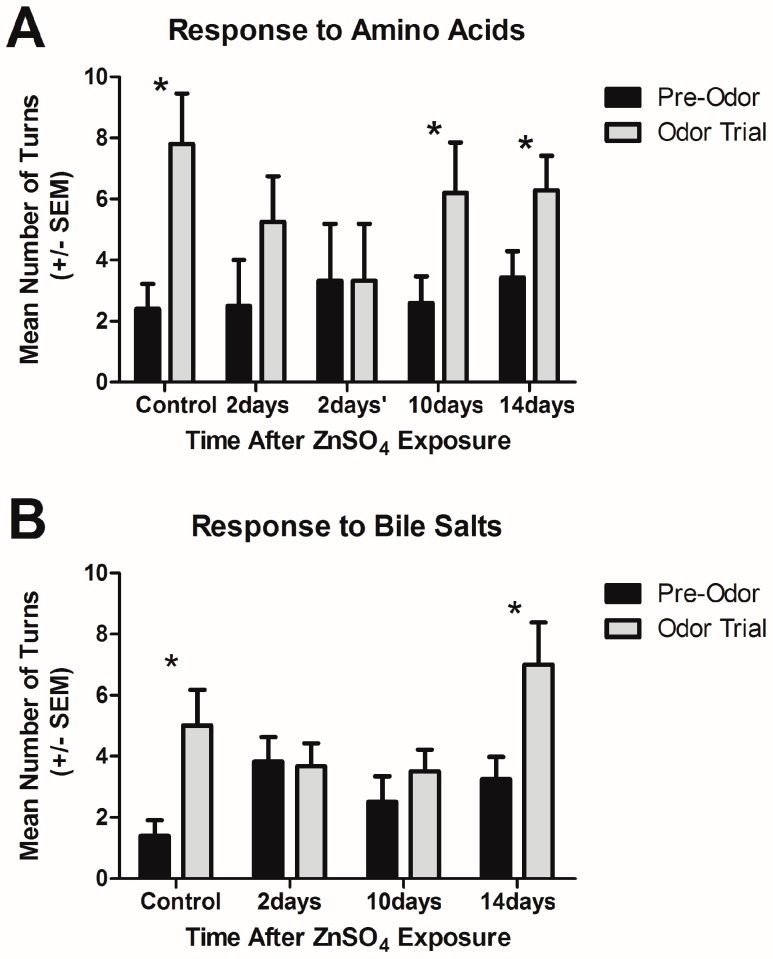
Behavioral responses were compared before (pre-odor) and after (odor trial) delivery of an amino acid (**A**) or bile salt (**B**) mixture. Control fish made significantly more turns after exposure to either mixture. (**A**) At 2 days following zinc sulfate treatment, fish did not show a statistically significant response to amino acids; however, there appeared to be a subset of fish that showed some response to the odor (2 days) and others who exhibited no change in behavior in response to the odor (2 days’). By 10 and 14 days after chemical exposure, fish made more turns during the amino acid odor trial; (**B**) Two days after exposure to zinc sulfate, fish did not respond to the bile salts mixture. Even after 10 days, the response to bile salts was not different from the pre-odor behavior. However, when given 14 days following zinc sulfate exposure the ability to perceive bile salts was regained. * *p* < 0.05.

Unlesioned control fish significantly increased their number of turns following delivery of the amino acid mixture, displaying appetitive behavior (ZBC term 1.7) in response to this food stimulus (Figure 5A; *p* < 0.05; *n* = 5). Control fish also increased their number of turns after exposure to a bile salt mixture (Figure 5B; *p* < 0.05; *n* = 5), although they did not turn as much as they did after amino acid exposure. The fish appeared to exhibit a kin recognition response (ZBC term 1.89) to bile salts by often appearing to swim with their shadow, as reported previously [21]. An anosmic control with complete occlusion of both olfactory organs was performed in previous studies [20,21] demonstrating that olfaction, not gustation, was tested in this experimental paradigm.

At two days after exposure to zinc sulfate, variable responses to an amino acid mixture were observed, likely as a result of the variable olfactory organ morphologies described in the electron microscopy study. Four of the seven fish tested at this time point appeared to respond to the odor by making more turns during the odor trial than in the pre-odor period (2 days in Figure 5A), although there was not a significant increase compared to pre-odor turns (*p* > 0.05). The remaining three fish in this group did not respond to the odor by altering their turning behavior from the pre-odor period (2 days’ in Figure 5A). When fish were given 10 days (*n* = 5) following zinc sulfate exposure to recover, they once again exhibited a significant response to amino acids, and this response continued when tested at 14 days (*n* = 7) following zinc sulfate treatment (Figure 5A, *p* < 0.05).

The responses to a bile salts mixture indicate that loss of detection of this odor was more profound. At both two days (*n* = 6) and 10 days (*n* = 6) after zinc sulfate exposure, fish did not change their turning behavior after exposure to bile salts (Figure 5B, *p* > 0.05). Only after 14 days following zinc sulfate exposure were significant responses observed in response to bile salts (Figure 5B, *p* < 0.05, *n* = 8).

Therefore, following zinc sulfate exposure, there is a variable effect on the ability to sense amino acids: some fish exhibited no change in turning frequency compared to the pre-odor behavior, suggesting loss of the ability to sense the stimulus; others appeared to turn more during the odor trial, indicating their perception of the odor. By 10 days after intranasal infusion with zinc sulfate, all fish displayed increased turning frequency when exposed to the amino acid mixture. Perception of bile salts was lost at two days after zinc sulfate treatment and was not regained until 14 days following exposure. Thus, the effect on bile salt sensation was more affected by the toxicant than amino acid sensation.

## 3. Discussion

Our results demonstrate that a single exposure to zinc sulfate has a rapid, degenerative effect on the sensory epithelium of the olfactory organ of adult zebrafish, causing severe morphological changes in a concentration-dependent manner. The effects of sham treatments with salts (sodium sulfate, sodium chloride, and phosphate buffer) and sucrose solutions have been explored in other studies [5,19], and they found that osmotic pressure did not cause similar degeneration. Following intranasal infusion with 1 M zinc sulfate, there is initially inflammation of the sensory epithelium, reduction in sensory neurons, loss of cilia on the apical surface, and disruption of olfactory-mediated behavioral responses. When allowed a short recovery time following exposure, inflammation of the olfactory organ decreases, the sensory epithelium initially thins then regains anatomical resemblance to control tissue, and the fish regain perception of biologically relevant odors.

The effects of zinc on the olfactory system have been well documents in other animals. Humans treated with zinc sulfate as a prophylaxis for polio [28,29] and zinc gluconate nasal spray for alleviation of common cold symptoms [30] exhibited anosmia. It appears that zinc ions induce necrosis in olfactory tissues [31]. Zinc sulfate has a well-described destructive effect on the olfactory epithelium and olfactory perception in rodents [32,33,34,35]. The effects of zinc sulfate have also been explored in aquatic species, such as tadpoles [36,37] and catfish [5]. Ours is the first report of the specific effects of this toxicant on the morphology of the olfactory organ in the emerging model system of zebrafish.

There are progressive morphological changes in the olfactory epithelium of adult zebrafish after exposure to zinc sulfate. Dramatic inflammation is observed at two days, with the epithelium appearing swollen and lamellae often fused and indistinguishable. These observations are consistent with the inflammation observed after exposure to mercuric chloride in a carp [26]. Large vacuoles were observed throughout the swollen tissue and are likely either a proliferation of goblet cells as reported after copper exposure in rainbow trout [38] or proliferation of mucous cells shown following cadmium exposure in snakehead fish [39]. By five days following zinc sulfate exposure, inflammation of the olfactory organ had decreased and the anatomical structure began to resemble control tissue, with distinct lamellae and noticeably thinner epithelium. This thinned epithelium was similar to observations of the effects of Triton X-100 exposure in zebrafish [19]. Typical morphology of the olfactory organ was restored following 10 days recovery after zinc sulfate exposure, with the organ regaining a control appearance in shape and size. The recovery of the zebrafish olfactory system is remarkably faster than other models treated with zinc sulfate such as rodents, where restoration of control epithelial morphology takes at least 30 days [33], but is similar to the regeneration time course seen in catfish [5].

We have shown previously that exposure to a detergent causes similar effects to those reported here. In that study, we found that detergent lesions removed the apical regions of the epithelium, with immature and basal cells remaining [19]. In addition, there is an increase in cellular proliferation in the sensory epithelium. Since some cells survive the detergent exposure and mitosis is enhanced, the epithelium is rapidly restored. The time course in that study closely resembles our current findings with zinc sulfate exposure. The rapid recovery is likely due to reasons described by Cancalon [40] who found that following lesioning of the olfactory nerve in garfish, there is a rapid phase of regeneration from immature sensory neurons that were spared followed by replenishment of the sensory epithelium from neurogenesis of basal cells.

Analysis of the ultrastructure of the apical surface of the epithelium revealed that zinc sulfate exposure led to the loss of ciliated structures with the retention of microvilli. Scanning electron microscopy studies showed there is a rapid loss of sensory cilia as early as two days following zinc sulfate exposure, while microvilli in the sensory epithelium and non-sensory cilia and generally remain intact. The loss of sensory cilia and the preservation of microvilli and non-sensory cilia have been observed in another fish species following exposure to mercuric chloride [26], suggesting the possibility of a specific response to heavy metals that results in retention of some cell types or surface structures and loss of others. Differential responses of sensory neuron subtypes to other types of damage have been reported elsewhere. Both chronic and acute application of Triton X-100 causes more significant atrophy of ciliated OSNs than microvillous OSNs [20,21,24]. In addition, olfactory nerve transection results in more rapid degeneration of ciliated OSNs than microvillous OSNs in the trout [41].

A few specimens showed a greater extent of damage, with complete loss of both cilia and microvilli and only microridges remaining on the apical surface, similar to those described following mercury exposure in a carp [26]. Cells of the zebrafish epidermis and non-sensory epithelium possess these microridges [11], perhaps suggesting that the extensive damage of the chemical lesion nearly ablated the sensory epithelium leaving only supporting cells and non-sensory epithelium. Ciliated structures in the sensory epithelium began to reappear as early as five days following exposure, though they appeared shorter, thinner, and more sparsely distributed than in control tissue. It was not until 10 days following toxicant exposure that cilia in the sensory epithelium were observed in densities and dimensions that appeared similar to control levels.

Behavior assays were performed to determine the potential effects of zinc sulfate on olfactory perception. Major odorants for zebrafish are amino acids, detected by microvillous OSNs and initiating feeding behavior, and bile salts, detected by ciliated OSNs and indicating social cues. The behavior assay, in which the number of turns fish made in the 30 s before and 30 s after odor exposure, showed a loss of perception of both bile salts and amino acids immediately following zinc sulfate exposure, and a mixed response to amino acids where some fish showed no reaction to an amino acid mixture and others turned more frequently following that stimulus. It is likely that the responders possess functional microvilli, as we saw with many specimens in the ultrastructural analysis, and the non-responders have lost both cilia and microvilli, such as the specimens with only microridges remaining. When given 10 days to recover following a single treatment of zinc sulfate, fish regained the ability to detect amino acids, while the perception of bile salts was still absent. Only when allotted 14 days following zinc sulfate exposure did fish respond to bile salts with a significant increase in turning frequency. Although sensory epithelium regeneration was evident by 10 days following exposure, the behavioral response lags behind the morphological recovery. A likely explanation for this is that even though sensory neurons are present at 10 days, the synaptic connections and molecular machinery involved in olfaction are not intact at that time and take longer to establish olfactory perception. Thus, following zinc sulfate exposure the recovery of apical surface ultrastructure followed by function of microvillous OSNs precedes the respective recovery of ciliated OSNs. This is similar to the response shown in previous studies through both chronic and single applications of detergent. Following Triton X-100 exposure, the ability to detect amino acids is retained, while the ability to sense bile salts is not [20,21].

It is interesting to speculate that microvillous OSNs mediating the feeding response are protected or that ciliated OSNs involved in social behaviors are more susceptible to damage. Since the olfactory organ is in direct contact with the environment and is exposed to potentially harmful substances, the olfactory epithelium has developed a number of defense mechanisms including persistent neurogenesis [42], detoxification processes [43,44,45], heat shock proteins [46,47], antioxidants [48,49], and structural barriers [50,51]. Any or all of these mechanisms could be involved in the retention of amino-acid sensing microvillous OSNs after zinc sulfate exposure.

Several studies have demonstrated that retention of olfactory-mediated food-finding behavior is often primary over olfactory-mediated social or reproductive preservation. Utilizing methyl bromide-induced olfactory lesions, rats exhibited responses to odorants indicating feeding behavior at three days following exposure [52]. When goldfish are exposed to copper, the perception of bile salts, prostaglandins, and steroids is more affected than the ability to smell amino acids [53]. Salmon exposed to cadmium show reduced freeze responses to skin extract, but there was no effect on avoidance behavior [54]. Since the skin extract is a complex mixture of odorants, it may be possible that this is due to differential effects on OSNs. Both chronic and acute treatment of the olfactory organs with Triton X-100 eliminates the response to bile salts, but perception of amino acids remains [20,21]. However, copper toxicity reduces electro-olfactogram responses to both a bile salt and an amino acid mixture [55]. This repeated pattern may suggest an evolutionary process to preserve one’s immediate needs over olfactory-mediated social and predatory cues.

We demonstrate here that the olfactory epithelium can rapidly recover after zinc sulfate insult. Exposure to zinc sulfate results in a temporary loss of ciliated OSNs and their associated behaviors with the preservation of microvillous OSNs. The complete degeneration and regeneration of olfactory organ structure and function happen within 14 days. The effects of exposure to zinc sulfate are similar to the effects of detergent, although subtle differences exist, perhaps indicating that there is a universal response of olfactory neurons to chemicals. In sum, it appears that ciliated OSNs of zebrafish are more affected by toxic insult than microvillous OSNs.

## 4. Materials and Methods

### 4.1. Exposure to Zinc Sulfate

Adult zebrafish, *Danio rerio*, of both sexes were obtained from a regional distributor. All fish were maintained in 10-gallon glass aquaria with aerated, conditioned water at 28.5 °C and fed twice daily with freshwater flake food. Care was taken to minimize animal suffering and limit the number of animals used, and all animal procedures were approved by the Institutional Animal Care and Use Committee (Animal Welfare Assurance #A3252-01; protocol 13-05-01, 5 August 2013).

Before exposure to zinc sulfate, fish were anesthetized in 0.03% MS222 (methane sulfonate salt, Sigma, St. Louis, MO, USA) until unresponsive to a tail pinch. Exposed fish received intranasal administration of 2 μL of 0.2, 0.5, 1, or 5 M zinc sulfate (Sigma, St. Louis, MO, USA) in dH_2_O to the right olfactory organ. A thin strip of Vaseline petroleum jelly (Unilever US, Englewood Cliffs, NJ, USA) was applied between the nostrils to prevent leakage to the left side, which was untreated to serve as an internal control. Fish were placed on ice to allow for a 3-min exposure to the zinc sulfate before being returned to a recovery tank and examined after 2, 3, 5, 7, 10, or 14 days.

### 4.2. Tissue Processing

Unlesioned control and chemically lesioned fish were euthanized by overexposure to 0.03% MS222. They were then placed in 4% paraformaldehyde for 24 h before length measurements and sex were recorded. Whole heads were dissected and decalcified with RDO rapid decalcifying solution (Electron Microscopy Sciences, Hatfield, PA, USA), dehydrated through ascending ethanol washes, and embedded in paraffin. Semi-serial, 10-μm sections in the horizontal plane were mounted on positively charged slides.

### 4.3. Histology and Immunohistochemistry

Some sections were stained following typical hematoxylin and eosin protocols to observe morphology. Other sections were labeled with immunohistochemistry following typical protocols. Briefly, sections were rehydrated and treated with 3% H_2_O_2_ in dH_2_O to remove endogenous peroxidases. Non-specific binding was blocked with 2% bovine serum albumin and 0.4% Triton X-100 in phosphate-buffered saline (PBS) for 1 h at room temperature. Slides were incubated in a humid chamber at 4 °C for 24 h in anti-calretinin (Santa Cruz Biotechnology, Dallas, TX, USA) at 1:1000 made in blocking solution. Following PBS rinses, sections were incubated at room temperature for 1 h in biotinylated anti-goat IgG (Vector Laboratories, Burlingame, CA, USA) at 1:100 made in blocking solution. Following PBS rinses, sections were treated with ABC solution (Vector Laboratories) for 1.5 h and exposed to diaminobenzidine solution (Vector Laboratories) until sufficient staining was observed. Sections were dehydrated and coverslipped before viewing.

### 4.4. Optical Density Measurements

Using SPOT Software 5.0 (Diagnostic Instruments, Sterling Heights, MI, USA), images were taken of anti-calretinin labeled sections at 20× and converted to 8-bit gray scale. Using ImageJ software (National Institutes of Health, Bethesda, MD, USA), the amount of antibody labeling was estimated by calculating the optical density of staining. To do this, the gray area intensity of the sensory area of three olfactory lamellae per section from three alternating semi-serial sections of each fish was measured and averaged. The background gray area intensity was also measured, and the optical density for that fish was calculated using the formula: OD = log (background intensity/average intensity of measured regions). Comparisons within groups were made using 2-way analysis of variance with Bonferroni post hoc analysis, comparisons of percent difference between right and left olfactory organs across groups were made using 1-way analysis of variance with Tukey’s post hoc analysis, and comparison to zero was made using a *t*-test. A significance level of 0.05 was used. A sample size of 3 fish was used at each time point.

### 4.5. Scanning Electron Microscopy

Control and chemically lesioned fish were euthanized with an overdose of 0.03% MS222 at 2 days, 5 days, and 10 days time points (*n* = 3–5 for each group). Whole fish were fixed in 3% glutaraldehyde in PBS at 4 °C for 48 h. Fish were decapitated, and heads were rinsed in PBS before undergoing a secondary fixation in 1% osmium tetroxide in PBS at room temperature for 1 h. Heads were then dehydrated through ascending ethanol rinses and Hexamethyldisilazane (Electron Microscopy Sciences, Hatfield, PA, USA) before being left to air-dry for 24 h. Olfactory organs were dissected, mounted, sputtered with gold, and imaged with a Hitachi S-4500 scanning electron microscope (Hitachi High Technologies, Schaumburg, IL, USA).

### 4.6. Odor-Driven Behavior Assay

Treatment groups for behavioral testing included untreated control fish and fish 2 days, 10 days, and 14 days following zinc sulfate exposure. All fish were age matched and equal numbers of male and female fish were used. Treated groups were exposed to 1 M zinc sulfate as described in Section 4.1; however, both olfactory organs were exposed to zinc sulfate in order to examine the effects on olfactory acuity.

Olfactory responses were observed using a behavioral assay as previously described [20,21]. Fish were placed individually into testing tanks on the appropriate day following exposure to zinc sulfate. They were allowed 1.5 h to acclimate to the tank between all trials. A digital video recorder was set above the testing site to capture behavioral data. For each trial, fish were exposed to an amino acids mixture (alanine, cysteine, histidine, methionine, and valine; 10 mM each, all from Sigma) or bile salts mixture (taurocholic acid, taurodeoxycholic acid, taurochenodexoycholate, lithocholic acid, glycocholic acid, and glycochenodeoxycholate; 10 mM each, all from Sigma). Odorants were delivered through a tube on one side of the testing apparatus while water was simultaneously delivered through another tube on the opposing side. For each trial, which tube delivered an odorant or water was determined with a random number generator. Behavior was assessed 30 s prior to odorant delivery and 30 s following delivery. Sample sizes ranged from 5 to 8 fish for each group. Each fish was tested for a response to amino acids and bile salts, with the tank rinsed and refilled and the fish re-acclimated between trials. For some control fish (*n* = 6), a trial of water was delivered simultaneously on both sides of the test apparatus as a mechanical control. Behavior trials were recorded, and swimming behavior was analyzed by observing the number of turns fish made pre-odorant delivery (pre-trial) and post-odorant delivery (trial). For each treatment group, data was averaged and comparisons of pre-trial and trial were made using a two-way analysis of variance and Tukey’s post hoc test. A significance level of 0.05 was used.
